# Management of Six Root Canals in Mandibular First Molar

**DOI:** 10.1155/2015/827070

**Published:** 2015-01-05

**Authors:** Claudio Maniglia-Ferreira, Fabio de Almeida Gomes, Bruno Carvalho Sousa

**Affiliations:** ^1^Department of Endodontics, University of Fortaleza (UNIFOR), 60811-905 Fortaleza, CE, Brazil; ^2^Department of Endodontics, Federal University of Ceará (UFC), 62010-560 Sobral, CE, Brazil

## Abstract

Success in root canal treatment is achieved after thorough cleaning, shaping, and obturation of the root canal system. This clinical case describes conventional root canal treatment of an unusual mandibular first molar with six root canals. The prognosis for endodontic treatment in teeth with abnormal morphology is unfavorable if the clinician fails to recognize extra root canals.

## 1. Introduction

The goal of root canal treatment is to clean the root canal system as thoroughly as possible and to fill it in all its dimensions to eliminate or at least reduce the microbial load in the canals [1–5]. Therefore, the details of unusual root canal morphology should be known to ensure successful root canal treatment [6–11]. The canals should be accurately located, cleaned, shaped, and obturated [12–14].

Internal complexities of the root canal are genetically determined and described in many reports on root canal morphology of different populations [9, 15–20]. Also, with ageing, secondary dentine deposition forms partitions and extensive differentiations of the root canal system, which result in separate canals and transverse connecting systems [21].

The mandibular first molar usually has three or four canals [22, 23]. The mesial root has two canals with an isthmus in between [18, 19, 22, 24]. This system may have an accessory mesial canal at a prevalence that ranges from 0% to 17% [25, 26]. Kottor et al. [11] and Ahmed et al. [18] found a prevalence rate of 4% and 3% for 3 canals in mesial and distal roots. Therefore, this occurrence in the same tooth is rare [27, 28].

When a preoperative radiograph reveals an atypical tooth shape and an unusual contour, further radiographs at different angles, if necessary, and cone-beam computed tomography scans should be taken to confirm any unusual anatomical features [29]. Apart from these presentations, a wide variation of root and canal configurations of the maxillary first molars has been documented in the dental literature. Case reports of mandibular first molars presenting with five or more root canals are summarized in Table 1.

This clinical report describes the unusual morphology of a mandibular first molar with two roots and six root canals detected during routine root canal treatment.

## 2. Case Presentation

A 28-year-old man was referred for root canal treatment of his left mandibular first molar. The clinical diagnosis was necrotic pulp. History revealed pain during mastication. Neither fistulae nor edema was observed in the soft tissue. There was no pain or tenderness to palpation, tooth mobility was within physiological limits, and gingival attachment was normal. The tooth was tender to vertical percussion. Thermal pulp testing (Endo-Frost, Coltène-Whaledent, Langenau, Germany) elicited a negative response.

**Figure 1:**
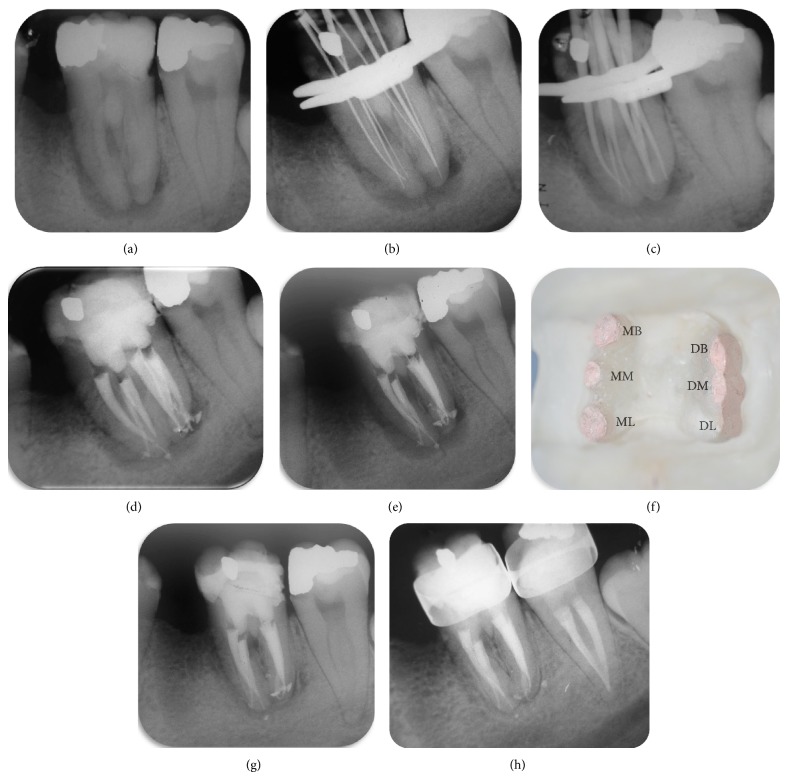
Preoperative radiograph shows normal root canal anatomy and apical lesion (a); radiograph to determine working length (b); radiograph shows gutta-percha cones placed in canals (c); postobturation radiograph shows 6 root canals and independent apical foramina ((d) and (e)); clinical view of pulp chamber after root canal treatment (f); periapical repair 6 (g) and 18 months after treatment (h).

The tooth was anesthetized using the standard inferior alveolar nerve blocks with 1.8 mL of 4% articaine and 1 : 100.000 epinephrine (Articaine, DFL Ind e Com Ltda., Rio de Janeiro, Brazil). Pretreatment radiographs of the tooth showed a normal root canal anatomy and an apical lesion (Figure 1(a)). After placing a rubber dam and disinfecting the area with 2% chlorhexidine gluconate, conventional coronal access was performed. Initial examination with an endodontic explorer revealed that the pulp chamber had four orifices, two mesial and two distal (MB, ML, DB, and DL). Accurate inspection, after exploring and cleaning the grooves, showed that there were middle mesial and middle distal canals located between the root canals previously identified. The canals were negotiated using a number 10 K-file (Dentsply-Maillefer, Ballaigues, Switzerland); an independent apical foramen was found. Irrigation was performed using 2.5% sodium hypochlorite solution (NaOCl) and 17% EDTA. Cleaning and shaping were performed using a reciprocation motion with the R25 Reciproc instrument (VDW, Munich, Germany) adapted to a Reciproc Silver motor (VDW, Munich, Germany).

The canals were negotiated to the working length (WL), as indicated by an apex locator (Root ZX locator, J. Morita MFG Corp, Kyoto, Japan), with a size 10 K-type file, confirmed radiographically (Figure 1(b)). The Reciproc R25 instrument was introduced into the canal until resistance was felt and then activated in reciprocating motion. The instrument was moved in apical direction using an in-and-out pecking motion of about 3 mm in amplitude with a light apical pressure. After three pecking motions, the instrument was removed from the canal and cleaned. Next, a size 15 K-type file was taken to the WL to check whether the canal was patent. These procedures of Reciproc use followed by patency check with the size 15 K-type file were repeated until the WL was reached by the Reciproc instrument for all root canals. After preparation was complete, the canals were rinsed with 5 mL of 17% EDTA, followed by 10 mL of 2.5% NaOCl [8].

Obturation was performed using the continuous wave of condensation technique and gutta-percha points (Odous, Belo Horizonte, Brazil) and Grossman sealer (Endofill, Dentsply, Rio de Janeiro, Brazil) (Figures 1(c), 1(d), and 1(e)). The conventional triangular access was modified to a trapezoidal shape to improve access to the additional canals (Figure 1(f)). The tooth received a permanent restoration, and the patient was seen six months later for follow-up. Clinical examination of the tooth was normal and the radiograph revealed decreased apical radiolucency (Figure 1(g)), despite the defective restoration present on coronal portion. Patient was referred for the placement of new crowns and during the 18-month follow-up, it can be noted that patient is under orthodontic treatment and complete healing of periapical lesion was achieved (Figure 1(h)).

## 3. Discussion

The main objective of root canal treatment is the thorough mechanical and chemical cleansing of the entire pulp cavity and its complete obturation with an inert filling material [11]. The most common reason for endodontic failure is infection that extends along the root canal system into the apical area [5]. Special attention should be paid to unfilled canal(s), because they are the least resistant path to leakage or reinfection [1, 15, 30].

Possible variations in the internal anatomy of human teeth should be known to ensure the success of endodontic treatment. The mandibular first molar usually has (i) a mesial root with 2 canals (94.4%); (ii) a third canal (middle mesial), at an incidence of 2.3%; (iii) a distal root with 1 (62.7%) or 2 (37.3%) canals [23].

Karapinar-Kazandag et al. [26] found that the prevalence of accessory mesial canals in the mesial root of mandibular molars is 18%; therefore, all negotiated accessory mesial canals were confluent with the main canals (MB or ML) and no canal ended in an independent apical foramen. Ryan et al. [27] reported a mandibular first molar with 3 separate mesial and 3 separate distal canals, but the distal root canals presented a single apical foramen. In our clinical case all the 6 canals had an independent apical foramen.

The recent development of technologies for endodontic treatment has focused largely on improving the quality of treatments [8, 26]. The introduction of apical locators, nickel-titanium (NiTi) mechanical instruments, operating microscopes, digital radiography, and cone-beam computed tomography greatly improved the ability to detect, clean, and shape root canals [15, 26, 29, 31–33]. Despite the disadvantages of not using magnification and an operating microscope in this clinical case, the middle mesial and middle distal canals were found during accurate inspection of grooves and isthmus between the root canals identified initially without magnification [26].

Many canals can be shaped in just a few minutes, although they may not be cleaned [8, 34]. Susin et al. [34] demonstrated that one of the greatest challenges is cleaning and disinfecting the connections between canals and isthmuses. Failure to access, debride, and disinfect this complex anatomy might have a direct effect on treatment outcomes, particularly in cases such as the one described in this study, in which all canals had an independent apical foramen [23].

The expression “shaping and cleaning” is intended to emphasize that canals are generally shaped first and then cleaned if irrigation protocols are followed [8, 35]. Shaping is critical, not only for effective cleaning, but also for three-dimensional obturation [31]. The canals in our case were three-dimensionally obturated using the continuous wave of condensation technique and medium nonstandardized cones. Grossman sealer was used because it penetrates into dentinal tubules well and serves as filler for root canal irregularities and for minor discrepancies between the root canal wall and core filling material [1]. It may also contribute to control microbial infection should there be microorganisms left on the canal walls or in the tubules [36]. The treatment was performed in a single session due to absence of clinical signs and symptoms of acute apical inflammation (pain, swelling, and apical exudate) [37].

From a clinical standpoint, radiographs or other imaging resources provide clinicians with the most appropriate method to detect variations in both root and canal anatomy. However, only by correct clinical examination and interpretation of these images can the clinician detect variations and be aware of them before and during endodontic procedures [32, 38]. Clearly, third mesial and distal canals in mandibular molars should be investigated and identified when planning root canal therapy [30, 39, 40]. The mesial and distal grooves of mandibular molars should be explored and cleaned. This might aid in the detection of a possible middle canal.

## 4. Conclusion

This clinical case report describes the endodontic treatment of a mandibular first molar with 6 root canals. Mandibular first molars with six root canals are rare, but each case should be carefully investigated clinically and radiographically to detect any anatomic anomalies. Possible variations in the internal anatomy of human teeth should be known to ensure successful endodontic treatment. Also, accessory canals in mandibular molars should be detected and negotiated to provide access for irrigation and filling materials into otherwise inaccessible isthmus.

## Figures and Tables

**Table 1:** Case reports on mandibular first molar.

Reference	Number of canals	Root canal configuration	
		Mesial root	Distal root
Demirbuga et al., 2013 [33]	5	3	2
Ryan et al., 2011 [27]	6	3	3
Kottoor et al., 2010 [41]	5	3	2
Yesilsoy et al., 2009 [30]	5	3	2
Chandra et al., 2009 [14]	5	2	3
Barletta et al., 2008 [13]	5	2	3
Fleming et al., 2010 [37]	5	2	3
Alves et al., 2012 [8]	6	3	3
Lee et al., 2006 [38]	7	4	3
Ricucci, 1997 [12]	5	3	2
Reeh, 1998 [39]	6	3	3
